# Dermoscopic Features of Lichen Amyloidosis in Caucasians—A Case Series and Literature Review

**DOI:** 10.3390/medicina57101027

**Published:** 2021-09-27

**Authors:** Magdalena Żychowska, Karolina Pięta, Izabela Rudy, Aleksandra Skubisz, Adam Reich

**Affiliations:** Department of Dermatology, Institute of Medical Sciences, Medical College, Rzeszow University, 35-310 Rzeszów, Poland; karolinapieta10@gmail.com (K.P.); izabelarudy10@gmail.com (I.R.); olaskubiszek@gmail.com (A.S.); adi_medicalis@go2.pl (A.R.)

**Keywords:** dermoscopy, dermatoscopy, primary cutaneous amyloidosis, lichen amyloidosis, macular amyloidosis, nodular amyloidosis, videodermoscopy, skin of color

## Abstract

Primary cutaneous amyloidosis (PCA) is characterized by the extracellular deposition of amyloid in the skin without systemic involvement. It comprises several clinical variants, the most common of which are macular amyloidosis (MA) and lichen amyloidosis (LA). PCA is frequently observed in Asians, while it is considered to be very rare in Caucasians. In the latter population, the condition often poses a diagnostic challenge. Dermoscopy has already been proved to be a useful, non-invasive diagnostic tool in various non-neoplastic skin diseases. In the paper, we present three Caucasian patients (skin phototypes I–II) with histologically confirmed LA. Under dermoscopy, central white hubs with grayish-brown dots and globules were observed in all three cases. Vascular structures were present in two cases and had the morphology of red globules and thick, unfocused branching lines intersecting the white hubs. A comprehensive review of the literature retrieved twelve papers presenting the dermoscopic features of PCA, including five articles on the dermoscopy of LA. The vast majority of these studies have been conducted on the Asian population, and there is a lack of data on the dermoscopic findings for patients with skin type I or II. The literature review revealed that MA and LA share several dermoscopic similarities (the presence of a white central hub and grayish dots), but also display distinct features. Compared to the dermoscopic features of LA in darker skin phototypes, our patients presented less pronounced pigmentation and more evident vascular structures. Nevertheless, further studies are needed in order to reliably evaluate the dermoscopic features of PCA in various ethnicities.

## 1. Introduction

Primary cutaneous amyloidosis (PCA) is a common condition in the Asian and South American populations, characterized by the extracellular deposition of amyloid fibrils in the skin without internal organ involvement [1,2,3,4,5,6,7,8,9,10,11,12]. The disease may be associated with itch and disfigurement, and therefore, it may lead to significant psychological distress [1,2].

PCA comprises several clinical variants, including macular amyloidosis (MA), lichen amyloidosis (LA), nodular amyloidosis (NA), biphasic amyloidosis (BA, a combination of MA and LA), amyloidosis cutis dyschromica (ACD), and anosacral amyloidosis [2,3]. LA is the most common subtype of PCA, clinically presenting as pruritic hyperpigmented and hyperkeratotic papules with a predilection for lower legs [1]. MA is characterized by the presence of brown macules arranged in a rippling pattern. NA is associated with the local proliferation of plasma cells, which are the source of amyloid light chain L protein deposits in the skin, and clinically manifests with nodular lesions [4,5]. ACD is an extremely rare subtype of PCA, characterized by dotted and reticular hyperpigmentation and hypopigmented spots, which usually start to develop during childhood [6].

Dermoscopy of PCA has been the subject of very few studies so far [1,2,3,4,5,6,7,8,9,10,11,12]. The majority of papers present the dermoscopic features in the Asian and South American populations [1,2,3,6,8,9,10,11,12]. As the condition is infrequent in Europe, data on the dermoscopic findings in this population are scarce [7]. In fact, there is a lack of studies reporting the dermoscopic features of PCA in patients with Fitzpatrick skin type I or II. Some authors have postulated that dermoscopic findings may vary depending on the patients’ ethnicity [8,9,10]. In the paper, we present three Caucasian patients (skin type I–II) with LA and discuss the dermoscopic characteristics. We also performed a literature review on the dermoscopy of PCA.

## 2. Case Reports

**Figure 1:**
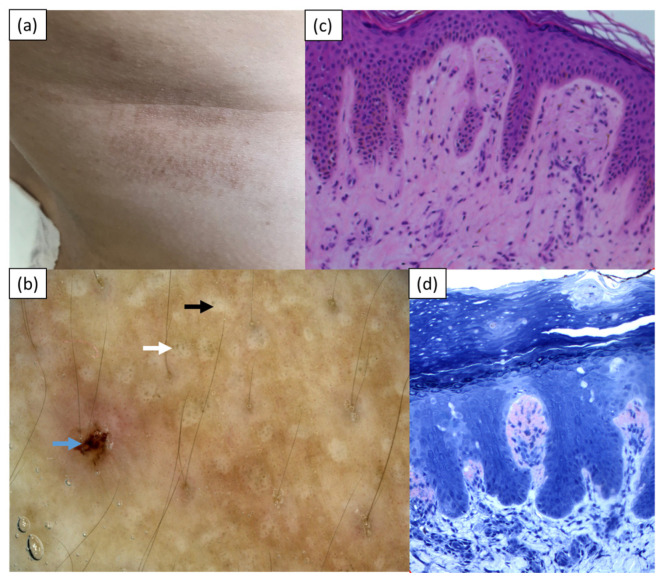
Case 1—(**a**) brown coalescing papules on the right side of the trunk; (**b**) videodermoscopy showing central white hubs (black arrow) with greyish dots (white arrow) and a single erosion (blue arrow); (**c**) histopathological examination showing hyperkeratotic epidermis, pigment incontinence, and deposits of eosinophilic material in the papillary dermis (hematoxylin & eosin); (**d**) deposits in the papillary dermis staining positive with Congo red.

Case 1 was a 29-year-old Caucasian woman (skin phototype I/II) who was referred to the Department of Dermatology with pruritic papular lesions on the trunk and upper extremities with a 4-year duration. The skin lesions were initially treated with topical glucocorticosteroids, with temporary improvement. Upon admission, numerous hyperpigmented coalescing papules were present on the back and, to a lesser degree, on the chest and upper extremities (Figure 1a). A basic laboratory work-up, chest X-ray, and ultrasound of the abdomen did not reveal any abnormalities. Videodermoscopy (Canfield D200^EVO^, Canfield Scientific GmbH, Bielefeld, Germany) showed white central hubs with grayish-brown pigment dots in the center and at the periphery of the hubs, and small erosions (Figure 1b). A histopathological examination displayed hyperkeratotic epidermis, mild inflammatory infiltrate composed of lymphocytes, histiocytes, and granulocytes in the upper dermis, and deposits of eosinophilic material in the papillary dermis, which stained positive with Congo red (Figure 1c,d). The clinical presentation and histopathology were consistent with the diagnosis of LA. Topical treatment with 0.1% mometasone furoate ointment was started and resulted in a significant reduction in pruritus. Shortly thereafter, the patient stopped treatment because of her pregnancy and was lost for further follow-up.

**Figure 2:**
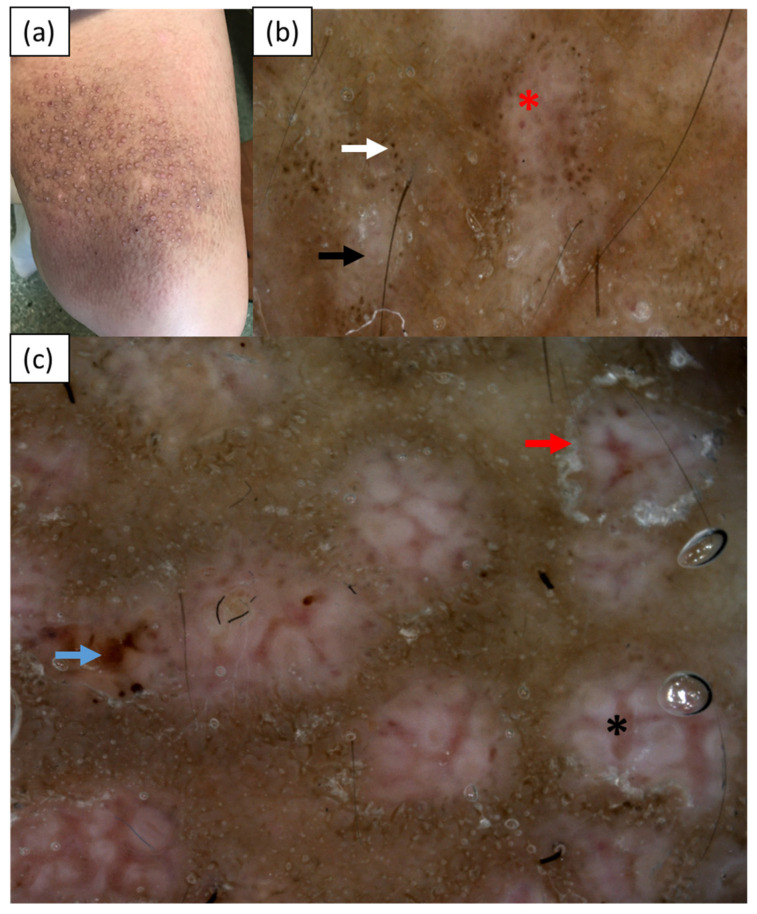
Case 2—(**a**) hyperkeratotic brown papules on the extensor surface of the left thigh; (**b**,**c**) videodermoscopy (Canfield D200^EVO^) showing central white hubs (black arrow) with greyish-brown globules at the periphery (white arrow), erosion (blue arrow) and white collarette scaling (red arrow). Red globules (red asterisk) in the center of the white hub and thick, unfocused branching vessels intersecting the white hubs (black asterisk) are present.

Case 2 was a 50-year-old Caucasian woman (skin phototype II) admitted to the Department of Dermatology for evaluation of disseminated hyperpigmented papules and nodules that had been gradually increasing in number for over 30 years. The skin lesions were accompanied by intense pruritus. Topical glucocorticosteroids and PUVA-therapy had been used in the treatment so far. In addition, the patient suffered from type 2 diabetes. Upon admission, multiple firm papules and nodules were present on the upper and lower extremities, abdomen, back, and buttocks (Figure 2a). Videodermoscopy (Canfield D200^EVO^, Canfield Scientific GmbH, Bielefeld, Germany) showed central white hubs on a grayish-brown background, brown dots and globules at the periphery, a white collarette scaling surrounding some of the white hubs, and erosions. Vascular structures in the form of red globules and thick unfocused branching vessels intersecting the central hubs were observed as well (Figure 2b,c). A histopathological examination revealed hyperkeratosis, acanthotic epidermis, vessels with thickened walls in the dermis, chronic perivascular infiltrate composed of histiocytes and lymphocytes, pigment incontinence, and single melanophages. Deposits of an eosinophilic substance that stained positive with Congo red were present in the papillary dermis. The histopathological image was consistent with the diagnosis of LA.

**Figure 3:**
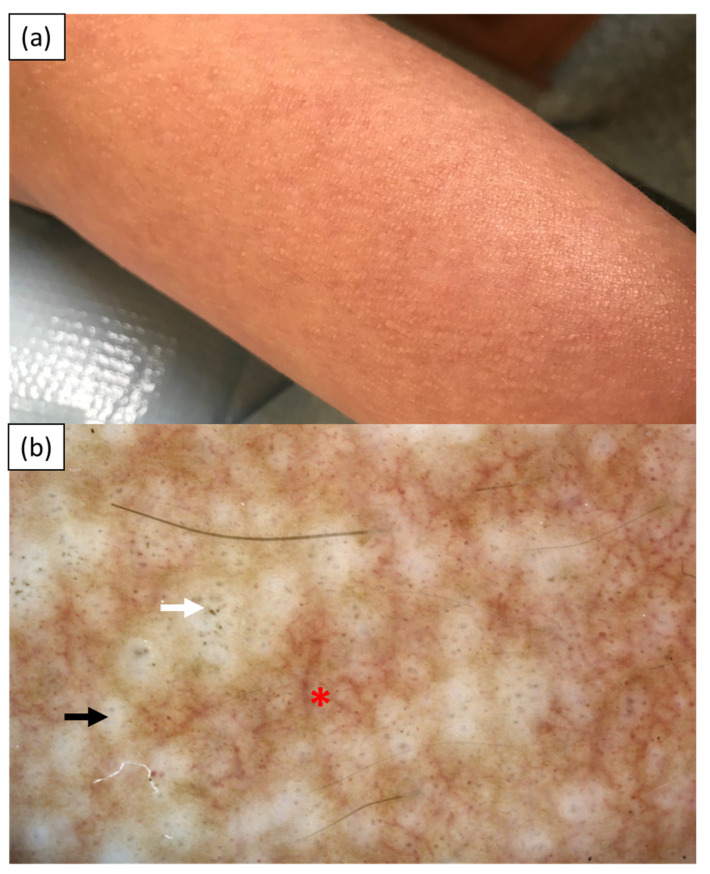
Case 3—(**a**) discrete skin-colored papules on the left forearm; (**b**) videodermoscopy (Canfield D200^EVO^) showing central white hubs arranged in a linear manner (black arrow) with greyish dots (white arrow) and branching and dotted vessels in between (red asterisk).

Case 3 was a 35-year-old Caucasian woman (skin phototype I/II) referred to the Department of Dermatology for evaluation of multiple severely pruritic papules on the limbs with an 8-year duration. Her comorbidities included the prediabetic state. Upon admission, multiple pinpoint skin-colored papules were observed on the upper and lower extremities (Figure 3a). Videodermoscopy (Canfield D200^EVO^, Canfield Scientific GmbH, Bielefeld, Germany) revealed central white hubs arranged in a linear manner with grayish dots and globules in the center and at the periphery. Numerous dotted, globular, and linear vessels were present between the white hubs (Figure 3b). Histopathology showed hyperkeratosis, vessels with thickened walls in the dermis, pigment incontinence, and eosinophilic deposits, which stained positive with Congo red. The findings were consistent with the diagnosis of LA. Numerous treatment strategies were undertaken, including systemic prednisone up to 40 mg/day, cyclosporin A at the dose of 350 mg/day (5 mg/kg/day), PUVA-therapy, methotrexate at a dose of 20 mg/week, systemic antihistamines, topical glucocorticosteroids, and topical 8% capsaicin. None of these therapies implemented brought any long improvement.

## 3. Discussion

In the majority of cases, PCA may be diagnosed clinically. As the condition is relatively rare in Caucasians and may have atypical presentation, it may pose a diagnostic challenge. The differential diagnoses include lichen simplex chronicus, lichen planus pigmentosus, hypertrophic lichen planus, and prurigo nodularis [10]. Histopathology remains the gold standard in doubtful cases. Still, dermoscopy may serve as a useful non-invasive diagnostic tool.

To analyze the dermoscopic findings in different variants of PCA, we performed a comprehensive search of three medical databases (PubMed, Web of Science, and Scopus) using the search terms ‘dermoscopy’ or ‘dermatoscopy’ combined with ‘primary cutaneous amyloidosis’ or ‘macular amyloidosis’ or ‘lichen amyloidosis’ or ‘nodular amyloidosis’ or ‘biphasic amyloidosis’ or ‘amyloidosis cutis dyschromica’. In total, twelve articles, including three original studies and nine case reports, were identified [1,2,3,4,5,6,7,8,9,10,11,12]. The dermoscopic characteristics of PCA are summarized in Table 1.

In the majority of cases, LA presented with central white hubs or scar-like centers surrounded by varied pigmentation, mainly in the form of gray-brown dots, globules, or peppering [2,7,11,12]. Behera et al. [12] coined the term ‘two-zone pattern’ to describe this arrangement of dermoscopic structures. Other less frequent findings included shiny white streaks under polarized light, blue-grey ovoid nest-like areas, concentric structures, white scaling, and white collarettes [1,11,12].

MA showed white, brown, or mixed central hubs under dermoscopy as well [2,3,10,11,12]. Chuang et al. [2] observed peripheral pigmentation in the form of fine streaks, leaf-like extensions, or bulbous projections in all of the analyzed cases of MA (*n* = 18). Madarkar et al. [11] reported the presence of semicircular hyperpigmented structures in 10 out of 12 patients with MA. On the other hand, Behera et al. [12] observed pigment dots, globules, or peppering in all of the studied cases of MA (*n* = 18). The authors coined the term ‘jigsaw puzzle pattern’ to describe the arrangement of the central hubs and pigmented structures in MA under dermoscopy. This pattern was observed by the authors only in a single case of LA, in which the aforementioned ‘two-zone pattern’ was dominant.

Histopathologically, white central hubs correspond to amorphous deposits of amyloid in the papillary dermis. The pigmentation observed under dermoscopy is related to pigment incontinence, basal hyperpigmentation, and the presence of melanin granules within the deposits in the dermal papillae [6]. Some authors also postulated that the dermoscopic findings in PCA depend on the extent of hyperkeratosis and acanthosis rather than on the number of amyloid deposits [2]. Brown central hubs were due to basket-weave orthokeratosis and white central hubs were due to marked hyperkeratosis. On the other hand, a white scar-like center was found to correspond to compact orthohyperkeratosis with acanthosis [2].

Other clinical variants of PCA, apart from MA and LA, are extremely rare, and much less is known about their dermoscopic presentations. Our literature search retrieved three reports of dermoscopic findings in NA [4,5,9]. Interestingly, the dermoscopic pattern was distinct from that of MA or LA. Rongioletti et al. [4] observed linear and serpentine vessels over an orange-yellow background, which is similar to the dermoscopic image of granulomatous skin conditions. An orange hue was also observed by Ferreira et al. [9]. In addition, shiny white streaks were present under polarized light.

To briefly summarize the previous reports, it can be concluded that LA and MA share common dermoscopic features, including central hubs and pigmented structures. In LA, central hubs are predominantly white, which is presumably related to pronounced orthohyperkeratosis. In MA, the central hubs may be white, brown, or mixed. The pigmented structures are usually located at the periphery of the hubs and may appear as gray-brown dots, globules, peppering, fine streaks, leaf-like extensions, or bulbous projections. Vascular structures have been very rarely observed under dermoscopy within the MA or LA lesions. On the other hand, little is known about the dermoscopic presentation of NA. Single available case reports highlight the presence of orange hue under the dermoscopy of NA.

Some authors have postulated that the dermoscopic presentation of PCA may vary depending on the patients’ skin phototype [10,11]. Most of the data available so far come from studies carried out in patients of Indian or Taiwanese descent [1,2,10,11,12]. Considering very few reports on the dermoscopic aspects of PCA, it is difficult to draw reliable conclusions. Nevertheless, observations from the papers published so far point at more pronounced pigmentation and the presence of thicker and darker structures in patients with darker skin types. In our case series, central white hubs were the dominant dermoscopic feature in all three cases. Pigment structures were discrete, particularly in Case 1 and Case 3, and included greyish-brown dots and globules. Interestingly, vascular structures were observed under dermoscopy in two out of three cases. In Case 2, thick, unfocused branching vessels intersecting the white hubs were prominent and corresponded to dermal vessels with significantly thickened walls in the histopathology. In addition, red globules were present within some of the white hubs. In Case 3, numerous branching and dotted vessels were observed in between the white hubs. So far, vascular structures have been rarely reported in PCA. Behera et al. [12] observed red or purple dots and globules in 7 out of 30 cases of LA. As mentioned above, elongated and serpentine vessels were reported in NA [4]. Nevertheless, to the best of our knowledge, the presence of thick branching vessels intersecting the central hubs is a dermoscopic finding that has not been reported in any clinical variant of PCA before.

## 4. Conclusions

Dermoscopy may serve as a useful non-invasive diagnostic tool in doubtful cases of PCA. Dermoscopic features of LA in Caucasians consist of central white hubs surrounded by greyish-to-brown dots. The pigmentation is less pronounced and the vascular structures are more evident when compared to dermoscopic findings in patients with Fitzpatrick skin phototype III–V.

## Figures and Tables

**Table 1:** A summary of studies describing the dermoscopic features of primary cutaneous amyloidosis (PCA).

	Lichen Amyloidosis					
	Arnold et al., 2012 (India/NA) *n* = 2	Chuang et al., 2012 (Taiwan) *n* = 17	Moscarella et al., 2018 (Italy) *n* = 1	Madarkar et al., 2021 (India) *n* = 18	Behera et al., 2021 (India) *n* = 30	Current cases (Poland) *n* = 3
Type of examination	polarized	Polarized and nonpolarized	N/A	Contact polarized	Contact nonpolarized	Contact nonpolarized
Two-zone pattern	-	-	-	-	25	-
Jigsaw puzzle pattern	-	-	-	-	1	-
Ridge and groove pattern	-	-	-	-	4	-
Hub and spoke pattern	-	-	-	-	5	-
Central hub	-	-	-	-	-	3
-white	-	7	1	5	-	3
-brown	-	-	-	2	-	-
-white and brown	-	-	-	1	-	-
White scar-like center	-	10	-	10	-	-
White structureless areas	-	7	-	10	-	-
Pigment dots/globules/ peppering	-	3	1	-	30	3
-clustered	-	-	-	-	18	2
-discrete	-	-	-	-	23	1
-starbust	-	-	-	-	12	-
-perieccrine	-	-	-	-	3	-
Blue-grey ovoid nest-like area	-	-	-	-	5	-
Concentric structures	-	-	-	-	5	-
White keratotic areas	-	-	-	-	5	-
Rim of white collarette	-	3	-	present	-	1
Diffuse white scaling	-	-	-	present	-	-
Comedo-like opening	-	-	-	-	1	-
Milia-like cyst	-	-	-	-	1	-
Red/purple dots and globules	-	-	-	-	7	2
Thick branching vessels	-	-	-	-	-	2
Shiny white streaks	2	-	-	-	-	-
Rosettes	-	-	-	1	-	-
Grayish-brown background	-	-	-	present	-	1
‘Daily lily flower’ structures	-	-	-	present	-	-
Erosions	-	-	-	-	-	2
	**Macular Amyloidosis**					
	Chuang et al., 2012 (Taiwan) *n* = 18	Madarkar et al., 2021 (India) *n* = 12	Wang et al., 2021 (China) *n* = 1	Behera et al., 2021 (India) *n* = 18	Sonthalia et al., 2020 (India) *n* = 2	
Type of examination	Polarized and nonpolarized	Contact polarized	NA	Contact nonpolarized	polarized	
Jigsaw puzzle pattern	-	-	-	17	-	
Ridge and groove pattern	-	-	-	8	-	
Hub and spoke pattern	-	-	-	4	1	
Central hub	18	-	1	-	1	
-white	11	3	-	-	1	
-brown	4	8	-	-	1	
-white and brown	3	1	-	-	1	
Pigment dots/globules/ peppering	-	5	1	18	-	
-clustered	-	-	-	17	-	
-discrete	-	-	-	10	-	
-starbust	-	-	-	8	-	
-perieccrine	-	-	-	7	-	
Peripheral pigmentation (fine streaks/leaf-like extensions/bulbous projections)	18	-	-	-	2	
Semicircular hyperpigmented structures	-	10	-	-	-	
Blue-grey ovoid nest-like area	-	-	-	1	-	
‘daily lily flower’ structures	-	2	-	-	-	
Erythematous background	-	3	-	-	-	
Perifollicular depigmented halo	-	-	-	-	1	
Fine white scales	-	-	-	-	1	
White-to-off white colored intersecting lines	-	-	-	-	1	
white-to-light brown colored structureless areas	-	-	-	-	1	
	**Nodular Amyloidosis**					
	Rongioletti et al., 2016 (Italy) *n* = 1	Ferreira et al., 2019 (Brazil) *n* = 1	DI Meo et al., 2018 (Italy) *n* = 1			
Type of examination	polarized	polarized	NA			
Shiny white streaks	-	1	-			
Orange-yellowish homogenous background	1	-	-			
Yellow teardrop-like structures	-	-	1			
Orange-pink background	-	1	-			
Elongated teleangiectasias	1	-	-			
Serpentine teleangiectasias	1	-	-			
	**Biphasic amyloidosis**					
	Kaliyadan et al., 2019 (Saudi Arabia) *n* = 1	Wang et al., 2021 (China) *n* = 1				
Type of examination	polarized	NA				
Brown stripes	-	1				
Irregular brown center	-	1				
White central hub	1	1				
Dotted/globular pigmentation	1	1				
	**Amyloidosis Cutis Dyschromica**					
	Wang et al., 2019 (China) *n* = 1					
Type of examination	NA					
Elliptical/round white spots	1					
Dotted/globular pigmentation	1					
Reticular pigment spots	1					
Striped hypopigmented spots	1					
Monotonous hypopigmented spots	1					

## Data Availability

The data presented in this study are available on request from the corresponding author.
